# Nanomechanics of few-layer materials: do individual layers slide upon folding?

**DOI:** 10.3762/bjnano.11.162

**Published:** 2020-11-30

**Authors:** Ronaldo J C Batista, Rafael F Dias, Ana P M Barboza, Alan B de Oliveira, Taise M Manhabosco, Thiago R Gomes-Silva, Matheus J S Matos, Andreij C Gadelha, Cassiano Rabelo, Luiz G L Cançado, Ado Jorio, Hélio Chacham, Bernardo R A Neves

**Affiliations:** 1 Departamento de Física, Universidade Federal de Ouro Preto, 35400-000, Ouro Preto, MG, Brazil; 2 Departamento de Física, Universidade Federal de Viçosa, 36570-000, Viçosa, MG, Brazil; 3 Departamento de Física, Universidade Federal de Minas Gerais, 30123-970 Belo Horizonte, MG, Brazil

**Keywords:** analytical methods, atomic force microscopy (AFM), molecular dynamics (MD), Raman spectroscopy, nanostructured materials

## Abstract

Folds naturally appear on nanometrically thin materials, also called “2D materials”, after exfoliation, eventually creating folded edges across the resulting flakes. We investigate the adhesion and flexural properties of single-layered and multilayered 2D materials upon folding in the present work. This is accomplished by measuring and modeling mechanical properties of folded edges, which allows for the experimental determination of the bending stiffness (κ) of multilayered 2D materials as a function of the number of layers (*n*). In the case of talc, we obtain κ ∝ *n*^3^ for *n* ≥ 5, indicating no interlayer sliding upon folding, at least in this thickness range. In contrast, tip-enhanced Raman spectroscopy measurements on edges in folded graphene flakes, 14 layers thick, show no significant strain. This indicates that layers in graphene flakes, up to 5 nm thick, can still slip to relieve stress, showing the richness of the effect in 2D systems. The obtained interlayer adhesion energy for graphene (0.25 N/m) and talc (0.62 N/m) is in good agreement with recent experimental results and theoretical predictions. The obtained value for the adhesion energy of graphene on a silicon substrate is also in agreement with previous results.

## Introduction

Layered materials such as graphite, talc, and transition metal dichalcogenides (TMDs), held together by strong covalent bonds within layers and relatively weak van der Waals interlayer interactions, have been the primary source of 2D materials [1]. These 2D materials exhibit unusual behavior associated regarding their flexural and adhesive properties [2–8]. For instance, self-assembled folded flaps and nanoribbons of graphene form by spontaneous folding, sliding, and self-pealing from a substrate [4]. Negative dynamic compressibility occurs in several 2D materials due to the dynamical wrinkling of layers [8]. Also, 2D materials folded in 3D origami-like structures [7,9–10] can, in principle, exhibit a tunable negative thermal expansion coefficient [11]. In all those cases, the interlayer adhesion energy (α), the substrate adhesion energy (α_s_), and the bending stiffness (κ) govern folding, sliding, and wrinkling of 2D materials, which are ultimately responsible for those unusual kinds of behavior. α is intimately related to tribological properties of layered materials as it determines the interlayer slip, which is the dominant mechanism to relieve stress at van der Waals interfaces, leading to phenomena such as the change from plate-like to membrane-like shapes in graphene, hBN, and MoS_2_ bubbles [12] or the circumferential faceting of multi-walled carbon nanotubes [13–14]. The interlayer slip is also intimately related to the dependence of κ on the thickness or the number of layers of a 2D material, which, in the case of very thin 2D materials, may be very different from that obtained from classical theories [12]. The quantification and understanding of the structural/dynamic response of multilayered 2D materials upon bending is also an essential issue regarding technological applications, such as deformable electronics, flexible reinforcements for brittle biomedical implants [15], and ultralight resonators suited as transducers of extremely small force or mass changes [16]. It is important to emphasize that the quality factor of the resonator depends on its maximum resonant frequency, which is intrinsically related to the flexural properties of the employed 2D material. As an example of an application based on the folds of a 2D material, we mention the recent development of an electromechanical device based on a water-induced electromechanical response in suspended graphene atop a microfluidic channel. The resistivity of the graphene membrane rapidly decreases by approx. 25% upon water injection into the channel due to the reduction of the folds in the suspended graphene [17].

After the successful synthesis of graphene in 2004 [1], many other 2D materials have been produced [12,18–23]. The investigation of their bending stiffness as a function of thickness, interlayer adhesion energy, and adhesion energy on several substrates is far from complete. Besides, uncertainties in the measured values of α can be large. For instance, experimental values of α for graphite ranging from 0.12 up to 0.72 N/m have been reported [24–31]. Regarding the bending stiffness κ, experimental values have been obtained through radial deformations [32], lattice dynamics studies [33], deformations of suspended layers [2,6], and bubble profiles [12]. The values reported for the bending stiffness of a graphene monolayer vary from 0.8 to 10,000 eV [34]. Also, there is no consensus on how κ varies with the number of layers [34].

In this work, we present a method to obtain interlayer and 2D material/substrate adhesion energies and the bending stiffness of 2D materials by experimentally probing the mechanical response of folded edges to deformation. A folded edge is defined as an edge region of the 2D material where it folds over itself during the exfoliation process. Our method is based on AFM measurements of the geometry and mechanical response of folded edges, and on the fitting of the experimental data by an analytical continuum model parameterized solely by α, κ, and the total thickness *d* of the folded 2D material. In principle, the model describes any 2D material, and its predictions are corroborated by comparison with classical molecular dynamics simulations and to results of previous investigations on graphene and talc. Because folds naturally occur in flakes of varying thickness, corresponding to multilayers with a different number of primitive layers, the proposed method provides a direct way to investigate the bending stiffness of 2D materials as a function of the flake thickness (or, equivalently, the number of layers). In the case of talc, we obtain κ ∝ *h*^3^ for materials thicker than five layers, indicating no interlayer sliding upon folding, at least in this thickness range. This result implies that layers in folds might be either compressed or stretched, leading to changes in their vibrational properties relative to a flat flake.

## Results and Discussion

**Figure 1:**
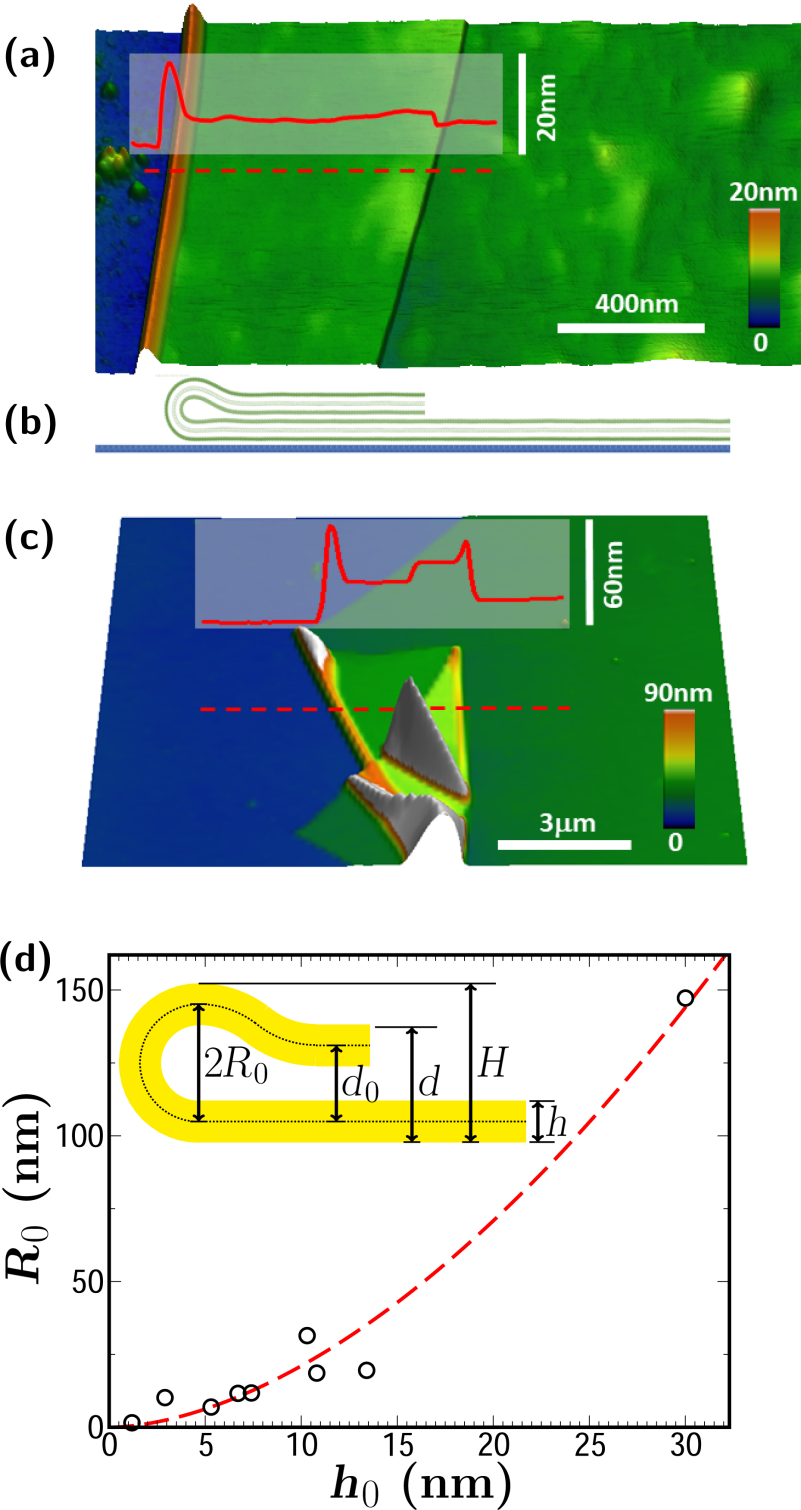
(a) AFM image of a folded edge of a talc flake (green-orange shades) with a thickness of approximately 2.4 nm (corresponding to two layers) deposited on a Si–SiO*_x_* substrate (blue shades). (b) Schematic drawing indicating the morphology of the fold shown in panel (a). (c) AFM image of an atypical fold with complex morphology eventually produced in the folding process. (d) Values of *R*_0_ for fold edges in talc flakes whose thickness is between 1 and 30 nm. In red, fitted curve *R*_0_ = *ah^b^*, where *b* = 1.75 and *a* = 0.38 (m^−0.75^). The inset shows a schematic drawing of a folded edge showing the relevant measured quantities *d*, *h* and *H*. *R*_0_ = (*H* − *h*)/2 and *d*_0_ = *d* − *h*, are parameters for the proposed continuum model.

A folded 2D material deposited on a substrate exhibits a cross-section geometry similar to that indicated in Figure 1 (see, for instance, Wang et al. [12] for electron microscopy images). Figure 1a shows an AFM image of a talc flake (green shades) with a thickness of approximately 2.4 nm (corresponding to two layers), which was exfoliated onto a silicon oxide substrate (blue shades). During the exfoliation/deposition processes, such a talc flake folds over itself, creating a well-defined folded edge, shown in orange shades. Figure 1b shows a schematic drawing of the morphology of the fold in Figure 1a. This is the morphology of the vast majority of folds investigated in this work. However, due to the random nature of the folding process, sometimes more complex folds are also produced, as those shown in Figure 1c. Some morphological parameters of the folds can be readily determined from the AFM line profiles, as shown by the red lines in Figure 1a and Figure 1c, such as its maximum height *H*, its total thickness *d*, and its layer thickness *h* (see the inset in Figure 1d for the definition of the parameters). Figure 1d shows the measured values of two of those parameters, *R*_0_ = (*H* − *h*)/2 and the flake thickness *h* for nine talc folds. The measured values of thickness *h*, from 1.2 to 30.0 nm, indicate that the measured folds involve materials from monolayer talc to approx. 30-layer talc. The corresponding values of the radius *R*_0_ range from 2.15 to 162 nm, that is, an increase of two orders of magnitude. The figure also shows a fitted curve *R*_0_ = *ah^b^*, where *b* = 1.75 and *a* = 0.38 (m*^−3/4^*).

**Figure 2:**
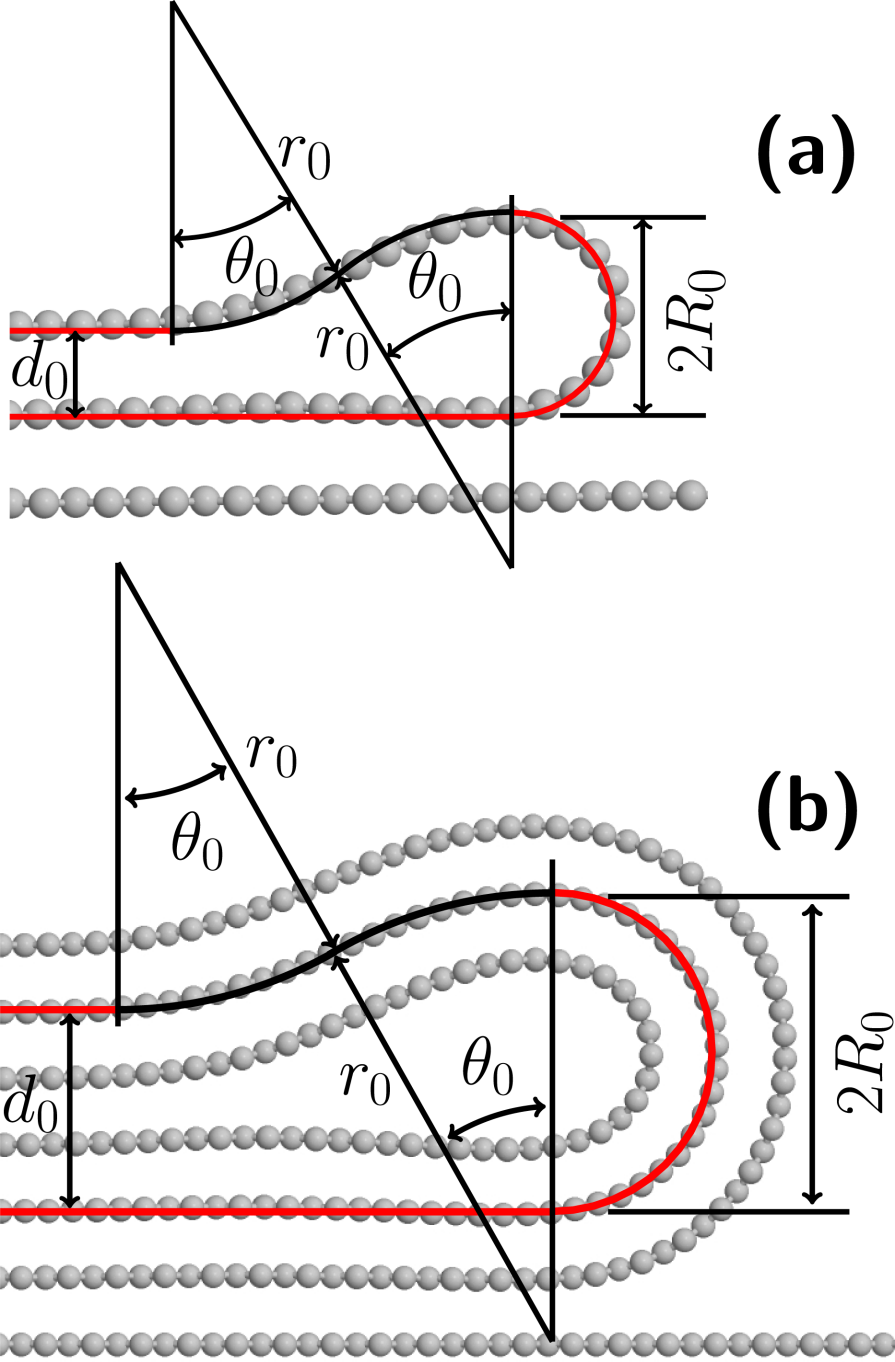
Carbon atom positions (gray circles) in cross sections of folded edges in (a) monolayer graphene and (b) three-layered graphene, as obtained through MD simulations. In both panels the red and black lines that superimpose the atomic positions depict the geometry of folded edges within our variational model, which consists of a sequence of straight lines and circular arcs with radii *R*_0_ (red arcs) and *r*_0_ (black arcs). In panel (a), the values of *R*_0_ and *r*_0_ are obtained through our model, Equation 1 and Equation 2, using experimental values of *d*_0_, κ and α. In panel (b), *R*_0_ and *r*_0_ were obtained through MD simulations.

To obtain κ and α from the AFM data, we propose a variational continuum model (see Supporting Information File 1, section “Deposited folded edges”) for the folded edges with the geometry depicted in Figure 2. This figure shows both cross-section geometries for folded edges in a graphene monolayer (Figure 2a) and in three-layered graphene (Figure 2b), obtained through MD simulations (details about MD simulations are found in Supporting Information File 1). As can be seen in Figure 2a, the model geometry consists of a sequence of straight lines and circular arcs with two possible radii, that is, the external radius *R*_0_ (red arcs) and the radius *r*_0_ of a half-soliton-like region (black arcs) [5]. Within our model, the concave arcs (up and down) of half-soliton-like region always have the same radius and length. Therefore, the model lines must pass the middle of the flake for folded edges in flakes more than one atom layer thick, as it is shown in the inset of Figure 1c and in Figure 2b for the three-layered folded edge. Figure 2 shows that the model geometry describes very well the morphology of folded edges in flakes with different thicknesses (the model lines nearly superimpose atomic positions in both panels of Figure 2). Within this model, mathematical relations between the geometrical parameters (*R*_0_, *r*_0_, and *d*_0_), and the adhesion (α) and flexural (κ) properties can be obtained variationally. The variational procedure within the model consists of the minimization of an energy functional that contains two terms, that is, the curvature energy *E*_c_ = ∫κ/(2*R*^2^)d*S* where *R* is the local curvature radius and κ is the bending stiffness. The adhesion energy *E*_a_ = α*S*_a_, where *S_a_* is the contact area and α is the adhesion energy per unit area between the 2D material and the precursor layered material. As a result of the variational procedure, we obtain (see Supporting Information File 1):

[1] $$r_{0}=\sqrt{\frac{3}{2}\frac{\kappa }{\alpha }}$$

and

[2] $$3\pi -\frac{3\pi }{2R_{0}^{2}}\left(\frac{\kappa }{\alpha }\right)+\frac{8}{\sqrt{2R_{0}-d}}{\left(\frac{3}{2}\frac{\kappa }{\alpha }\right)}^{1/4}=0.$$

Equation 1 and Equation 2 can be used to determine either the geometry of the folded edge from the properties of the 2D material (κ/α and thickness) or vice versa. In the particular case of the folded edge in a graphene monolayer shown in Figure 2a, we used literature values for α = 0.37 N/m [30] and κ = 0.231 aJ [35] to determine *r*_0_ and *R*_0_. In the case of the folded edge in three-layered graphene, we used values of *R*_0_ = 0.81 nm and *d*_0_ = 1.01 nm from MD simulations to obtain 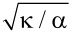 = 1.88 nm. Considering α the same for both folded edges of graphene, we thus found κ = 1.3 aJ for three-layered graphene, which is roughly six times the value reported for a graphene monolayer (κ = 0.231 aJ). Thus, MD results indicate that the change of κ with the number of layers in multilayered graphene is non-linear. The change of κ in a real 2D material will be discussed below.

**Figure 3:**
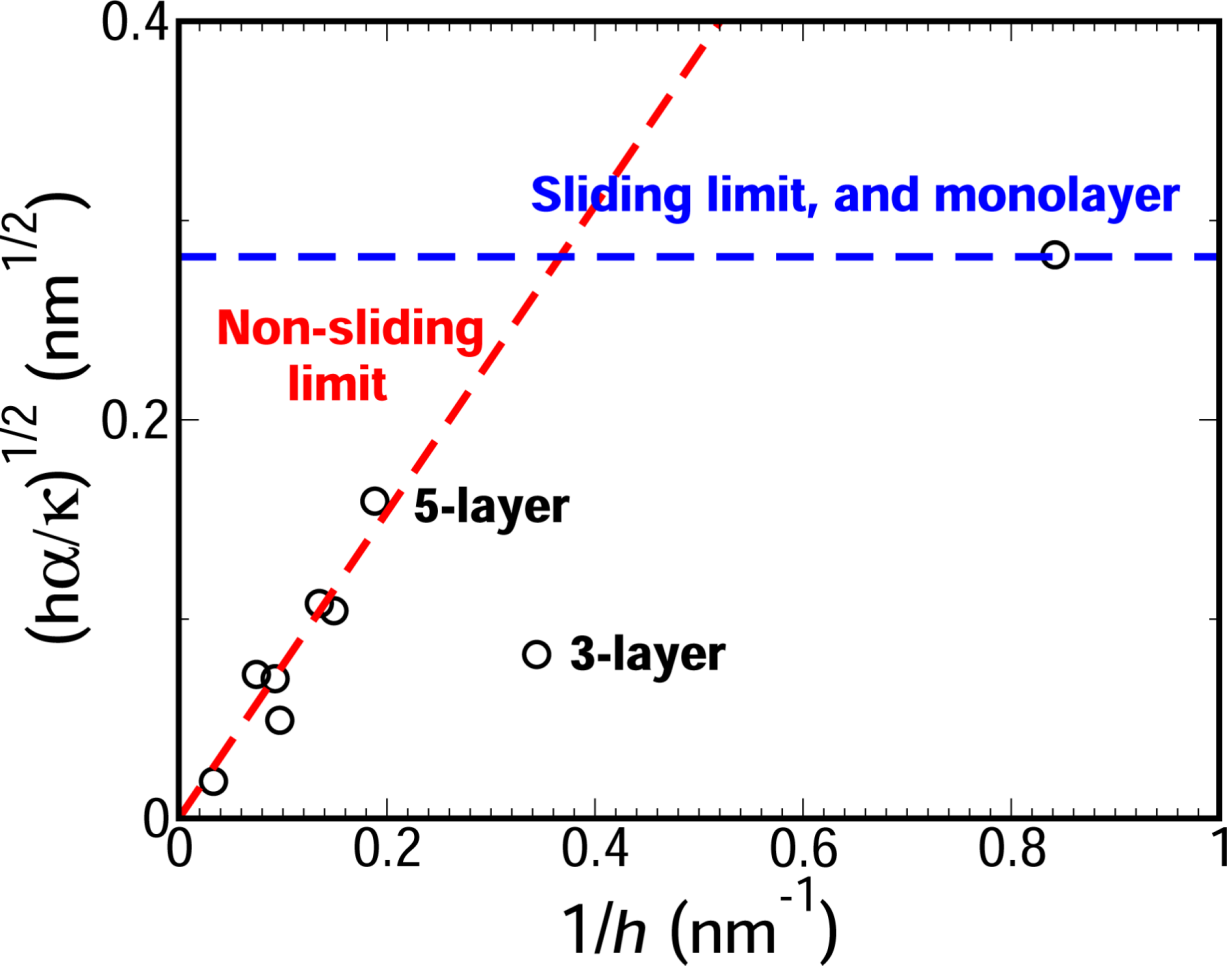
The value of 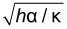 = 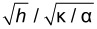 as a function of 1/*h* for the nine measured talc samples. *h* is a directly measured quantity (see Figure 1), and 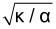 is obtained from Equation 2 with the measured values of *R*_0_ and *h*. The red and blue lines correspond, respectively, to the ideal non-sliding (κ ∝ *h*^3^) and sliding (κ ∝ *h*) limits. The values for the thinnest samples (monolayer, three-layers and five-layer talc) are explicitly indicated.

Equation 2 allows us to obtain the ratio κ/α for talc folds from the measured values of *R*_0_ and *d*_0_. Figure 3 shows the quantity *Q* = 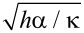 = 
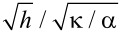
 as a function of 1/*h* for the nine measured talc samples. *h* is a directly measured quantity (see Figure 1), and 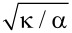, as mentioned above, is obtained from Equation 2. Assuming that α is constant for a given material, the behavior of *Q* as a function of *h*, *Q*(*h*), will solely depend on the behavior of κ as a function of *h*. In the limit of thick materials, we would expect that adjacent layers do not slide relative to each other upon an elastic bending deformation. In this non-sliding limit, we expect that κ ∝ *h*^3^, as predicted by the classical Euler–Bernoulli beam theory. In another limit, which we will call the sliding limit, we will assume the possibility that adjacent layers freely slide upon bending deformation. In this limit, which implicitly includes the monolayer case, we obtain κ ∝*h*. Both limits have been recently considered in the analysis of experimental profiles of bubbles in 2D materials [12]. In our present analysis, the functional form of *Q* leads to two asymptotic limits as a function of 1/*h*, that is, *Q* ∝ 1/*h* in the non-sliding limit and *Q* = constant in the sliding limit. Both limits are indicated in Figure 3 as red and blue lines fitted, respectively, to the seven thickest samples and the monolayer sample. Therefore, Figure 3 indicates that individual layers of multilayer talc with more than four layers do not slide upon folding. In contrast, we were not able to observe any sample behaving according to the proposed sliding limit, besides the (trivial, by definition) monolayer sample. There were no flakes with two or four layers in our samples. The sample with three layers shows a different behavior than the other samples and was much stiffer than expected. This might be attributed to a distinct morphology.

From the above, we conclude that sufficiently thick talc flakes behave like rigid objects without interlayer sliding. Therefore, upon fold formation, the layers at the inner part of the fold will have a compressive in-plane strain, and those at the outer part will have a tensile in-plane strain. The compressive and tensile strains could modify the vibrational properties of a fold compared to those of the (flat) bulk of the flake. This should be applicable to any 2D material and not restricted to talc. This hypothesis was investigated employing a near-field tip-enhanced Raman spectroscopy (TERS) setup [36–37] that can probe strain variations across the edge of a folded graphene flake of 5 nm thickness (see Supporting Information File 1, section “Near-field tip-enhanced Raman spectroscopy”). Our TERS measurements (see Supporting Information File 1) do not show significant changes in the G band of the Raman spectra in the folded region. This either indicates a negligible strain in the fold region, or a combination of small compressive and tesile stresses that keeps the frequency of the G band mostly unchanged. In either case, the Raman spectra suggest that at least partial sliding exists in the graphene fold, in contrast to the talc folds investigated above.

**Figure 4:**
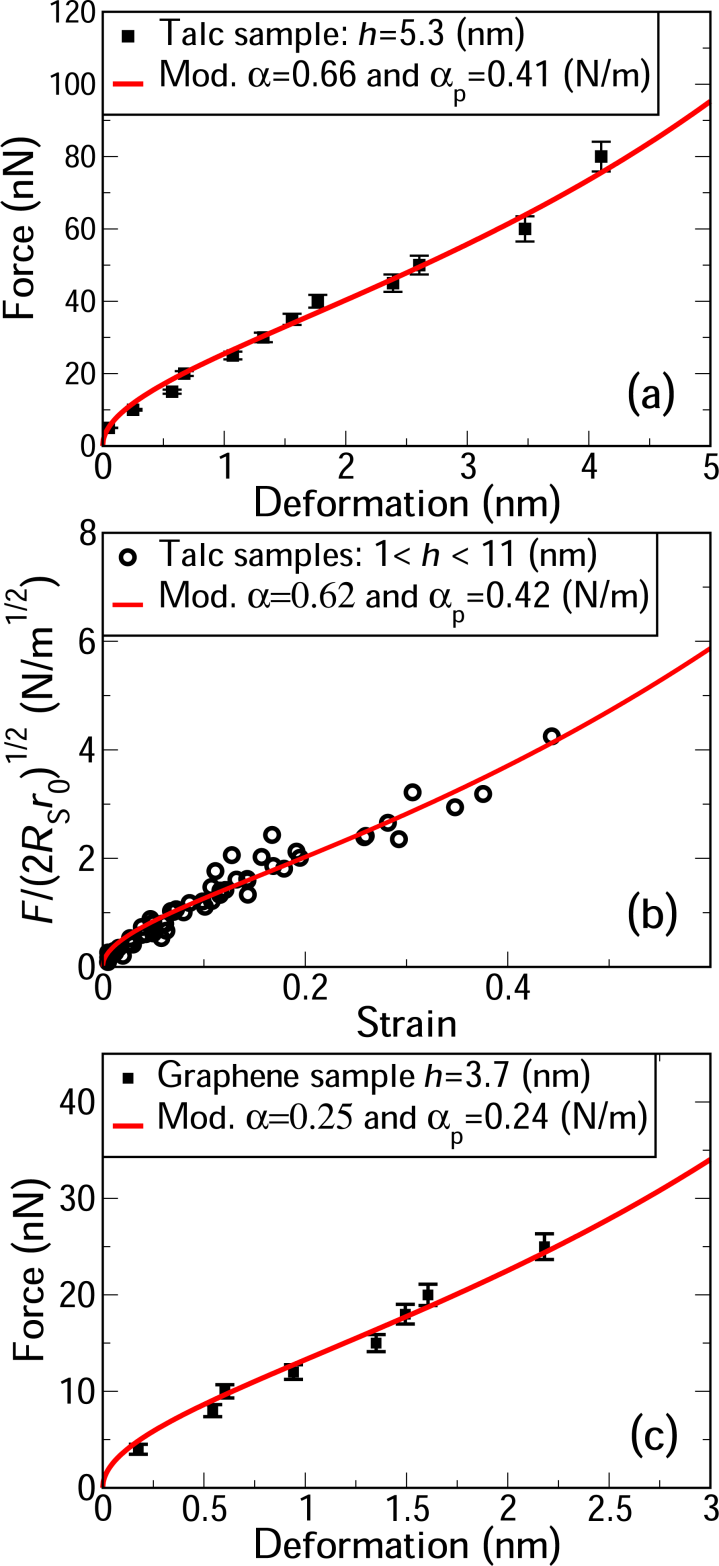
(a) Best fit of Equation 3 to forces and deformations measured using AFM on a 5.3 nm thick talc fold. (b) Best fit of Equation 3 to forces and deformations in folded edges of several talc flakes, with thicknesses of 1.2, 2.9, 5.3, 6.7, 7.4, and 10.9 nm. The vertical axis is the rescaled force, 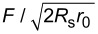 and the horizontal axis is the strain *S* = *D*/(2*R*_0_ − *d*) (see Supporting Information File 1). (c) Best fit of Equation 3 to AFM measurements on a fold in an eleven layers thick graphene flake.

We have so far addressed the ratio κ/α of talc folds obtained from Equation 2 with the measured values of *R*_0_ and *d* for uncompressed folds. As already discussed, the quantity κ/α allowed us to verify a prevalence of non-sliding behavior. However, one might be also interested in obtaining the absolute values of κ and α from the AFM measurements. In fact, to the best of our knowledge, no experimental measurement of α for talc has been reported so far. We will show that it is possible to obtain the value of α from AFM force-curve measurements on folded edges. According to our model (see Supporting Information File 1, section “Compressed folded edges”), when a spherical probe compresses a folded edge, the mechanical response is given by:

[3] $$\begin{array}{c} \frac{F\left(D\right)}{\sqrt{2R_{\text{s}}}}=\left[\left(\alpha_{\text{p}}-\alpha \right)\sqrt{\frac{2R_{0}-d}{16r_{0}}}-\left(\alpha +\alpha_{\text{p}}\right)\frac{\pi }{2}\right]\sqrt{D}\\ +\frac{2r_{0}^{2}\pi \alpha }{3}\left[\frac{\arctan\sqrt{\frac{D}{2R_{0}-D}}}{{\left(2R_{0}-D\right)}^{3/2}}+\frac{\sqrt{D}}{\left(2R_{0}-D\right)2R_{0}}\right]\\ +\left[\frac{\left(\alpha_{\text{p}}-\alpha \right)\left(2R_{0}-d-D\right)}{8\sqrt{r_{0}}}+\frac{4\alpha \sqrt{r_{0}}}{3}\right]\ln\left(\frac{\sqrt{2R_{0}-d}+\sqrt{D}}{\sqrt{2R_{0}-d}-\sqrt{D}}\right), \end{array}$$

where *D* is the deformation caused by the probe, *R*_s_ is the probe radius, and α_p_ is the adhesion energy per unit area between the 2D sample and the probe. Despite its length, Equation 3 contains only two adjustable parameters, that is, α and α_p_. The AFM height profiles provide *R*_0_ and *d*, while *r*_0_ can be obtained through Equation 1 and Equation 2. Figure 4a shows the fit of Equation 3 to the AFM force-vs-deformation measurements on a 5.3 nm thick talc fold. The obtained value of α = 0.60 N/m is consistent with the few theoretical results available (0.30 N/m [38] and 0.98 N/m [39]). Also, the obtained value of α_p_ = 0.42 N/m indicates that the interaction between talc and the silicon probe is smaller than the interaction between talc layers. To combine the data of several force-curve measurements in a single graph, we plotted the rescaled force 
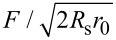
 as a function of the strain *D*/(2*R*_0_ − *d*) as shown in Figure 4b. The red curve in this figure represents our model (see Supporting Information File 1, Equation S13). The resulting values of α = 0.62 N/m and α_p_ = 0.40 N/m are similar to those obtained from the fit in Figure 4a, showing the consistency of these results.

Unlike in the case of talc, there are several results in the literature on the interlayer adhesion energy of graphene [26–31], providing good references to evaluate the α values predicted by our model. Figure 4c shows the best fit of Equation 3 to AFM measurements for a eleven layer thick graphene fold. To fit the experimental data, we used *R*_0_ = 4.5 nm and *d* = 5.25 nm, obtained from the height profile, and *r*_0_ = 11.7 nm, obtained through Equation 1 and Equation 2. The obtained value of α = 0.25 N/m is within the range of experimentally obtained values (0.12–0.72 N/m [24–31]). It is worth mentioning that our result (α = 0.25 N/m) compares well with other direct experimental determinations of α (0.27 N/m [28] and 0.37 N/m [30]), in which layers of graphene in highly oriented pyrolytic graphite were mechanically manipulated using a probe. Besides, the value obtained for the interaction between graphite and the silicon probe (α_p_ = 0.24 N/m) is consistent with the values reported in the literature (0.28 and 0.37 N/m [40–41]).

## Conclusion

We have shown that it is possible to obtain the interlayer adhesion energy and the bending stiffness of layered 2D materials by fitting results of AFM force curves on naturally occurring folded edges to an expression predicted by a simple model for those edges. The obtained interlayer adhesion energy for graphene (0.25 N/m) and talc (0.62 N/m) are in good agreement with recent experimental results [28,30] and theoretical predictions [34,38]. Besides, the value obtained for the interaction between graphene and the silicon probe (α_p_ = 0.24 N/m) is consistent with the values reported in the literature (0.28 and 0.37 N/m [40–41]). The proposed method also allows for the investigation of the dependence of the bending stiffness on the flake thickness. For talc flakes with a thickness equal or larger than 5.3 nm, we obtained a scaling relation (κ ∝ *h*^3^) consistent with the Euler–Bernoulli beam theory, indicating that layers in sufficiently thick flakes do not slip to relieve strain. In contrast, TERS measurements on edges in folded graphene flakes that were 5 nm, or 14 layers, thick shows no significant strain indicating that layers in graphene flakes up to 5 nm thickness can still slip to relieve stress. Even though it is applied here to homolayers, the present methodology could also bring invaluable insights about the interlayer interaction in the growing field of heterolayered 2D materials, probing the mechanical properties of typical interfaces such as graphene/hBN, graphene/TMDs, hBN/TMDs, or any other technologically relevant two-dimensional heterostructure.

## Supporting Information

Supporting information features the theoretical models for deposited and compressed folded edges, the experimental methods (including sample preparation, SPM characterization and near-field tip-enhanced Raman spectroscopy) and the computational details of the MD simulations.

File 1 Models, experimental part and computational details.
