# Supplementary material for: Nanomechanics of few-layer materials: do individual layers slide upon folding?
Source: Beilstein J Nanotechnol. 2020 Nov 30;11:1801–8. doi: 10.3762/bjnano.11.162 (PMC7722626; doi:10.3762/bjnano.11.162)
Supplement: File 1 — Models, experimental part and computational details. [file Beilstein_J_Nanotechnol-11-1801-s001.pdf]

BEILSTEIN JOURNAL OF NANOTECHNOLOGY

Supporting Information

for

Nanomechanics of few-layer materials: do individual layers slide upon folding?

Ronaldo J. C. Batista, Rafael F. Dias, Ana P. M. Barboza, Alan B. de Oliveira, Taise M. Manhabosco, Thiago R. Gomes-Silva, Matheus J. S. Matos, Andreij C. Gadelha, Cassiano Rabelo, Luiz G. L. Cançado, Ado Jorio, Hélio Chacham and Bernardo R. A. Neves

Beilstein J. Nanotechnol. **2020,** *11,* 1801–1808. doi:10.3762/bjnano.11.162

Models, experimental part and computational details

License and Terms: This is a supporting information file under the terms of the Creative Commons Attribution License (https://creativecommons.org/licenses/by/4.0). Please note that the reuse, redistribution and reproduction in particular requires that the author(s) and source are credited and that individual graphics may be subject to special legal provisions.
The license is subject to the *Beilstein Journal of Nanotechnology* terms and conditions: (https://www.beilstein-journals.org/bjnano/terms)

Contents

1 Theoretical Model **S2**

1.1 Deposited folded edges S2

1.2 Compressed folded edge S3

2 Materials and Methods **S8**

2.1 Sample Preparation S8

2.2 SPM characterization S8

2.3 Near-field tip-enhanced Raman spectroscopy S11

2.4 Molecular dynamics simulations S13

1 Theoretical Model

1.1 Deposited folded edges

We will consider a continuum model for the folded edges depicted in Figure 2 of the main manuscript, which shows folded edges in graphene obtained through MD simulations detailed below. We modeled such a folded edge as a half tube of radius R_0 connected to a structure we call "half soliton" (two arcs of radius r_0[1]). Within this model, we considered the following: (i) The folded edges are formed during the exfoliation process from material detached from the layered precursor, then the relevant quantity is the energy of adhesion instead of the energy of separation. (ii) We have considered as the reference (zero) energy the situation where the 2D material lies straight, deposited on a substrate. Thus, can write the energy to form folded edges containing two terms:

- $E_{\text{adhesion}} = \alpha L(\pi R_0 + 2\theta_0 r_0)$, which accounts for the energy cost to detach from a precursor layered material, the amount of 2D material required to form the folded edge, that is, ribbons of length L and widths πR_0 and $2\theta_0 r_0$.

- $E_{\text{bending}} = \int \frac{\kappa ds}{R_0^2} + 2\int \frac{\kappa ds'}{r_0^2} = L\frac{\kappa}{2}(\frac{\pi}{R_0} + \frac{2\theta}{r_0})$, which refers to the energy necessary to bend ribbons of length L and widths πR_0 and $2\theta r_0$ in the form or arc circles.

α is the adhesion energy per unit area between layers of the 2D material, and κ is the bending stiffness of the 2D material. Then, energy to form the folded edge per unit of length is:

$$\varepsilon = \frac{E_{\text{adhesion}} + E_{\text{bending}}}{L} = \alpha(\pi R_0 + 2\theta_0 r_0) + \frac{\kappa}{2}\left(\frac{\pi}{R_0} + \frac{2\theta}{r_0}\right). \tag{S1}$$

ε is a function of the variables R_0, r_0, and θ_0, which are all related through the expression $2R_0 - d = 2r_0(1 - \cos\theta_0) \approx r_0\theta^2$ (see Figure 2 of the main manuscript). Minimizing ε under the constraint of $g = 2R_0 - d - r_0\theta^2$, we obtain:

$$r_0 = \sqrt{\frac{3\kappa}{2\alpha}} \tag{S2}$$

and

$$3\pi - \frac{{r_0}^2\pi}{R_0^2} + \frac{8\sqrt{r_0}}{\sqrt{2R_0 - d}} = 0. \tag{S3}$$

1.2 Compressed folded edge

In this section, we considered that a spherical probe of radius R_s compresses the folded edge described in the previous section. To obtain an analytical expression for the force as a function of the deformation caused by the probe tip, we applied the following assumptions:

- Folded edges compressed by spherical probes have cross-section geometries similar to that described by the red, blue and black lines of the bottom panel of Figure S1, which also shows the geometry of compressed graphene folded edge obtained through MD simulations.
- The reference (zero) energy is that of the situation where the 2D material lies straight, deposited on another layer of the same 2D material, as shown in Figure 1 of the main manuscript for folded edges of talc.

- The relevant quantities are the energies of adhesion per unit of the area between layers (α) and between layer and probe (α_p) instead of the energy of separation.

Figure S1 shows that the curved parts of the folded edge are still very well described by a model containing half tubes and a half solitons. However, a flat region of width l appears between them, requiring an additional energetic contribution in Equation S1. Because the region l is contact with the probe, its contribution the energy is $(\alpha - \alpha_\mathrm{p})l$. Thus, the energy per unit of length of a compressed folded edge is the sum of adhesion and binding energies as follows:

$$\varepsilon = (\alpha - \alpha_\mathrm{p})l + \alpha(\pi R + 2\theta_0 r) + \frac{\kappa}{2}\left(\frac{\pi}{R} + \frac{2\theta}{r}\right). \tag{S4}$$

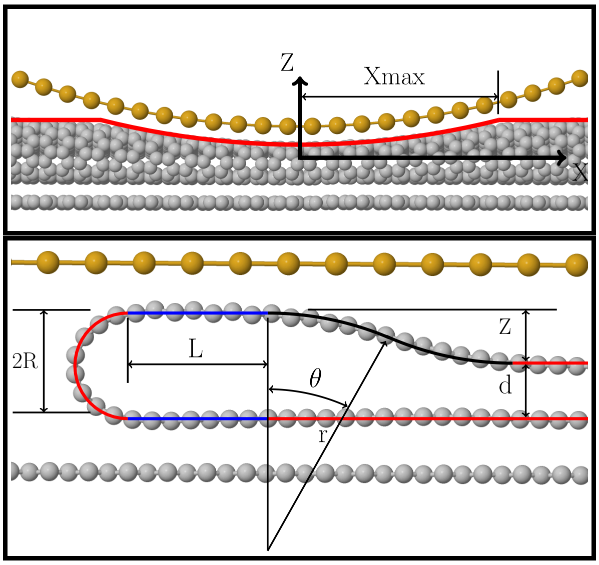

Figure S1: Molecular dynamics simulations of a folded edge in graphene (gray circles) deposited on a graphene substrate compressed by a hard cylinder of radius 10 nm (yellow circles). Top: front view of the compressed folded edge. Bottom: cross section of the folded edge shown in the top panel. The red, blue and black lines show our model for the cross-section geometry of the compressed folded edge.

The compression applied by the probe deposits the half soliton on the probe itself. Because the length of the half soliton ($2r_0\theta_0$) exceeds its horizontal projection ($2r_0 \sin\theta_0$), the difference $2r_0(\theta_0 - \sin\theta_0) - 2r(\theta - \sin\theta)$ contributes to the size of the blue flat regions in Figure S1. Another contribution,

$\pi(R_0 - R)$, comes from the reduction of the half-tube diameter relative to its uncompressed value (R_0). Thus, $2l = \pi(R_0 - R) + 2r_0(\theta_0 - \sin\theta_0) - 2r(\theta - \sin\theta)$ and energy can be rewritten as follows:

$$\varepsilon = (\alpha - \alpha_\mathrm{p})\left[\frac{\pi}{2}(R_0 - R) + r_0(\theta_0 - \sin\theta_0) - r(\theta - \sin\theta)\right] + \alpha\pi R + \frac{\kappa\pi}{2R} + 2\theta\left(\alpha r + \frac{\kappa}{2r}\right). \quad \text{(S5)}$$

Minimizing ε under the constraint of $g = 2R - d - r\theta^2$, we obtain $r \approx r_0 = \sqrt{\frac{3\kappa}{2\alpha}}$. (We considered $-\frac{(\alpha-\alpha_\mathrm{p})\theta^2}{12} + \alpha \approx \alpha$, which is reasonable because $(\alpha - \alpha_\mathrm{p})$ should be significantly smaller than α, and because $d > R_0$ for all folds, $\frac{\theta^2}{12} < \frac{R_0}{12r_0} = \frac{1}{12\sqrt{3}}$). Using $\kappa = \frac{2r_0^2\alpha}{3}$ and $\sin\theta \approx \theta - \frac{\theta^3}{3!}$ we obtain:

$$\varepsilon = (\alpha - \alpha_\mathrm{p})\left[\frac{\pi}{2}R_0 + r_0(\theta_0 - \sin\theta_0) - r_0\frac{\theta^3}{6}\right] + (\alpha + \alpha_\mathrm{p})\frac{\pi}{2}R + \frac{r_0^2\pi\alpha}{3R} + \frac{8\alpha\theta r_0}{3}. \quad \text{(S6)}$$

Defining $z = 2R - d$ and using $2R - d = 2r_0(1 - \cos\theta_0) \approx r\theta^2$ to eliminate θ ($\theta = -\sqrt{z/r_0}$), the above equation can be rewritten as follows:

$$\varepsilon = (\alpha-\alpha_\mathrm{p})[\frac{\pi}{2}R_0 + r_0(\theta_0 - \sin\theta_0) + \frac{z^{3/2}}{6\sqrt{r_0}}] + (\alpha+\alpha_\mathrm{p})\frac{\pi}{2}\frac{(z+d)}{2} + \frac{2r_0^2\pi\alpha}{3(z+d)} - \frac{8\alpha\sqrt{z}\sqrt{r_0}}{3}. \quad \text{(S7)}$$

We can write the force per unit length that the fold applies on the probe as a function of z as follows:

$$f(z) = -\frac{\mathrm{d}E}{\mathrm{d}z} = (\alpha_\mathrm{p} - \alpha)\frac{\sqrt{z}}{4\sqrt{r_0}} - (\alpha + \alpha_\mathrm{p})\frac{\pi}{4} + \frac{2r_0^2\pi\alpha}{3(z+d)^2} + \frac{4\alpha\sqrt{r_0}}{3\sqrt{z}}. \quad \text{(S8)}$$

From Equation S8, the force applied on a 3D probe by the folded edge can be obtained, provided that the probe profile over the length of the fold is known. Let us define such a direction as x, as shown in the upper panel of Figure S1. From here we can write:

$$F(h) = \int_{x_{\min}}^{x_{\max}} f(x)\mathrm{d}x, \tag{S9}$$

where $f(x)$ is force per unit length and h is a parameter that determines the probe height. For an object whose profile over the length of the fold is an arc circle (see the top panel of Figure S1), Equation S9 becomes:

$$F(h) = 2\int_{0}^{x_{\max}} f(x)\mathrm{d}x = 2\int_{h}^{2R_0-d} f(z)\frac{\mathrm{d}x}{\mathrm{d}z}\mathrm{d}z =$$

$$2\int_{h}^{2R_0-d} f(z)\frac{z-(R_{\mathrm{s}}-h)}{\sqrt{R_s^2-(z-R_s-h)^2}}\mathrm{d}z, \tag{S10}$$

where R_{s} is the probe radius and h and $2R_0 - d$ are the minimal and maximal values of z, respectively. Equation S10 can be rewritten as follows:

$$F(h) = 2\int_{h}^{2R_0-d} f(z)\frac{\mathrm{d}z}{\sqrt{\frac{R_{\mathrm{s}}^2}{(z-R_{\mathrm{s}}-h)^2}-1}} =$$

$$2\int_{h}^{2R_0-d} f(z)\frac{\mathrm{d}z}{\sqrt{(1+\frac{z-h}{R_{\mathrm{s}}}+(\frac{z-h}{R_s})^2+\ldots)^2-1}}$$

$$\approx 2\sqrt{\frac{R_s}{2}}\int_{h}^{2R_0-d}\frac{f(z)\mathrm{d}z}{\sqrt{z-h}}, \tag{S11}$$

where terms of the order $[(z-h)/R_s]^2$ and above are neglected because $z-h$ at most equals to deformations caused by the probe ($D = 2R_0 - d - h$), which are usually significantly smaller than R_s. Inserting Equation S8 in Equation S11 and solving the integrals we obtain:

$$F(D) = \sqrt{2R_s}\Bigg[\frac{-(\alpha+\alpha_p)\pi}{2}\sqrt{D} + \frac{2r_0^2\pi\alpha}{3}\left(\frac{\arctan\sqrt{\frac{D}{2R_0-D}}}{(2R_0-D)^{3/2}} + \frac{\sqrt{D}}{(2R_0-D)2R_0}\right) + \left(\frac{(\alpha_p-\alpha)(2R_0-d-D)}{8\sqrt{r_0}} + \frac{4\alpha\sqrt{r_0}}{3}\right) ln\left(\frac{\sqrt{2R_0-d}+\sqrt{D}}{\sqrt{2R_0-d}-\sqrt{D}}\right) + \frac{(\alpha_p-\alpha)}{4\sqrt{r_0}}\sqrt{2R_0-d}\sqrt{D}\Bigg]. \tag{S12}$$

R_0 and d can be determined from AFM height profiles and r_0 can be determined from Equation S3. Also, R_s is usually known in AFM experiments, which leaves two adjustable parameters in Equation S12, that is, α and α_p.

The force the compressed folded edge applies on the probe can be written as a function of the strain ($S = \frac{D}{2R_0-d}$) as follows:

$$\frac{F(S)}{\sqrt{2R_s}\sqrt{r_0}} = \frac{-(\alpha+\alpha_p)\pi}{2}\sqrt{\frac{2R_0-d}{r_0}}\sqrt{S} + \frac{(\alpha-\alpha_p)}{4}\frac{2R_0-d}{r_0}\sqrt{S} + \frac{2\pi\alpha}{3}\left(\frac{r_0}{2R_0}\right)^2\sqrt{\frac{2R_0-d}{r_0}}\left(2\sqrt{S} + \frac{8}{3}\left(1-\frac{d}{2R_0}\right)^{3/2}S^{3/2} + 3\left(1-\frac{d}{2R_0}\right)^{5/2}S^{5/2}\right) + \left(\frac{(\alpha_p-\alpha)(1-S)}{8}\frac{2R_0-d}{r_0} + \frac{4\alpha}{3}\right) ln\left(\frac{1+\sqrt{S}}{1-\sqrt{S}}\right), \tag{S13}$$

where we use $\arctan\left(\sqrt{\frac{D}{2R_0-D}}\right) \approx \sqrt{\frac{D}{2R_0-D}} - \frac{1}{3}\left(\frac{D}{2R_0-D}\right)^{3/2}$ and neglected terms of the order $(D/2R_0)^3$ and above in the expansion of $\frac{1}{1-D/2R_0}$. We also divided both sides of the above equation by $\sqrt{r_0}$. If the folded edges have the same proportions, that is, the same values of $\frac{r_0}{R_0}$ and $\frac{d}{r_0}$, a graph of $\frac{F(S)}{\sqrt{2R_s}\sqrt{r_0}}$ as a function of the strain will be similar for folded edges of different thickness. In fact, the bottom

panel of Figure 4 of the main manuscript shows that talc folds of thickness varying from 1 to 11 nm exhibit a similar dependence of $\frac{F(S)}{\sqrt{2R_s}\sqrt{r_0}}$ on the strain.

2 Materials and Methods

2.1 Sample Preparation

The graphene and talc samples were prepared by the mechanical exfoliation onto a 300 nm thick Si oxide layer covering the Si substrate. Folded layers were initially identified using optical microscopy and then scanned by AFM.

2.2 SPM characterization

In all results shown in this paper, NSC18/NoAl silicon cantilevers with typical spring constant $k \approx 2.8$ N/m, nominal radius of curvature $r \approx 10$ nm and resonant frequency $\omega_0 \approx 75$ kHz were employed. More accurate estimations of k and r were carried out by the use of the Sader's method [2] and by imaging reference samples, respectively. The AFM characterization was carried out on a Bruker MultiMode SPM using the Peak Force Quantitative Nano-Mechanical imaging mode® [3]. With the Peak Force QNM imaging mode® we can measure the maximum deformation of the sample, defined as the penetration of the tip into the surface at the peak force (highest force). As the load on the sample under the tip increases, the deformation also increases. The measured deformation may include both elastic and plastic contributions. We work in the elastic regime. The maximum sample deformation is calculated from the difference in separation from the point where the force is zero to the peak force point along the approach curve (see Figure S2) [3]. The hysteresis loop in Figure S2 is related to the dissipated energy during the peak-force loop (tip approach and retraction) [3].

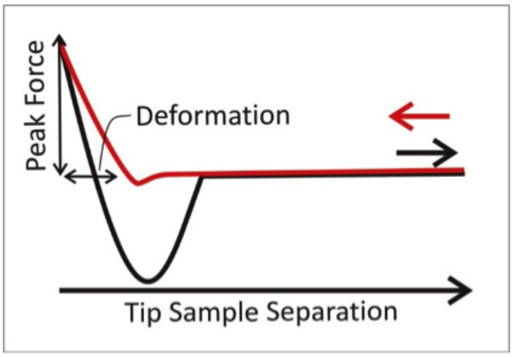

Figure S2: Schematic plot force versus tip–sample distance and the deformation value.

Figure S3a shows an AFM image of an atypical talc flake (green shades) with approximately ten layers, which was exfoliated onto a silicon oxide substrate (blue shades). During the exfoliation/deposition processes, such a talc flake folded back and forth over itself, creating a well-defined folded stripe, shown in yellow shades. Figure S3b shows a 3D perspective zoom of the dashed rectangle region in Figure S3a. The red dotted line schematically indicates the back-and-forth folding process, which yielded a double-edged folded stripe. We were able to infer this geometry through larger images, showing the whole flake (not shown). A careful inspection of line profiles taken in Figure S3b reveals that each edge of this double-fold has a different height. This is also corroborated by molecular dynamics simulations of a similar geometry, see Figure S3c, where the heights of each edge are slightly different.

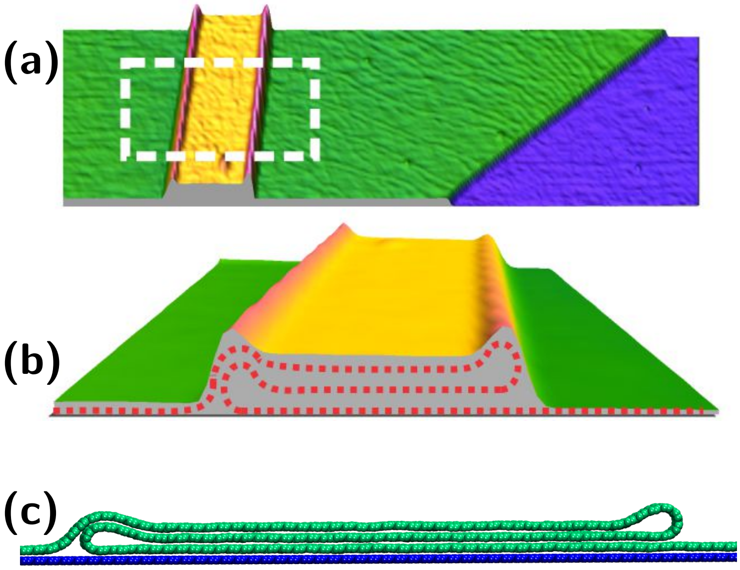

Figure S3: (a) AFM image of a double-folded edge in a talc flake (green-yellow shades) with approx. ten layers deposited on a Si–SiO_x substrate (blue shades). (b) Perspective view of the region inside the rectangle shown in panel (a). The dotted line in panel (b) is a guide to the eye, indicating the formation of the double-folded edges. (C) Morphology of a double-folded edge in graphene obtained through MD simulations.

Figure S4 shows the measured deformation as a function of the applied tip force for a fold and a flat region of the flake near the fold. As expected, the deformation on the flat surface is much smaller than on a fold. In other words, the fold clearly shows a totally different deformation mechanism, when compared to a flat surface.

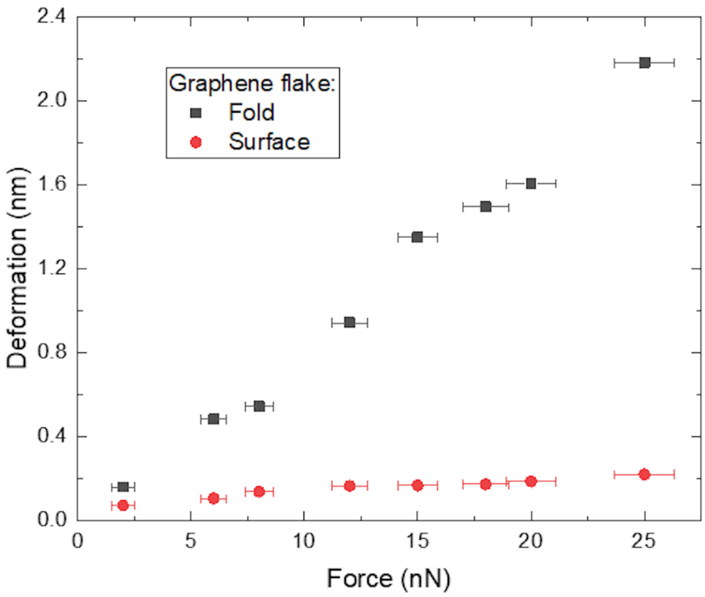

Figure S4: Deformation as a function of the applied tip force for a fold (black squares) and a flat region of the flake near the fold (red circles).

2.3 Near-field tip-enhanced Raman spectroscopy

For TERS measurements, we use a radially polarized laser beam with an excitation energy of 633 nm (1.96 eV) and a custom-built AFM scan head, working in a shear-force mode. The optical and AFM systems are coupled via gold plasmon-tuned tip pyramids (PTTP) [4] to get the tip-enhanced Raman signal. We use a laser power of 100 μW to avoid sample heating.

Figure S5a shows a near-field Raman map of the intensity of the G peak, where the blue and dark blue regions correspond to the folded graphene flake and the substrate, respectively. The dashed white line marks the location of the edge of the fold. Figure S5b shows a map of the Raman shift for the same fold. To detect such displacements clearly, we average the horizontal lines in Figure S5a and Figure S5b, and depict the result in Figure S5c, where we plot the relative Raman shift (red curve) and intensity (blue curve) of the G peak as a function of the lateral distance. The vertical dotted black line in this panel marks the position of the folded edge, where a change in the position of the G peak is also observed. It has been previously shown that strain in graphene causes displacements in the

G peak frequencies [5]. Compressive strain upshifts the G band and tensile strain downshifts the G band. The TERS results depicted in Figure S5c show a very mild change in the G band frequency, of the order of 0.5 cm^{-1}. Inspection of the G band linewidth also shows a mild change in the full width at half maximum (FWHM), which also increases by only 0.5 cm^{-1}. Considering the values larger than 10 cm^{-1} shift for 1% strain reported in the literature [5], our TERS results indicate that either no signiticant strain happens, or a combination of compressive and tensile strain occurs, keeping the G band frequency mostly unchanged. One could still wonder if the TERS signal is able to see the inner layers. Previous works show that ten graphene layers on a Si substrate reduce the Raman response of the substrate by 90% [6]. This picture is qualitatively right in our measurement, but it cannot be quantitatively utilized because we use a TERS configuration that is different. Nevertheless, for this specific flake, whose thickness is about 5 nm (determined by the TERS/AFM measurements), the measured TERS signal certainly contains important contributions from inner layers.

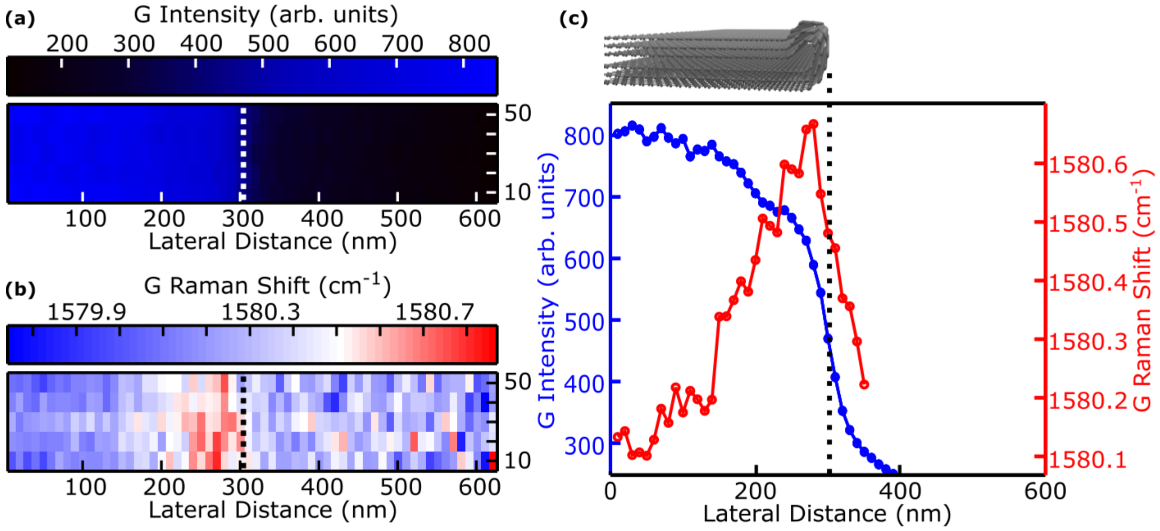

Figure S5: (a) Near-field Raman image of the intensity of the G peak as a function of the lateral distance, where the blue and dark blue regions correspond to the folded graphene flake and the substrate, respectively. The dashed white line marks the location of the edge of the fold. (b) Map of relative Raman shift, where blue corresponds to lower frequencies and red to higher frequencies. (c) Intensity (blue) and relative Raman shift (red) of the G peak as a function of the lateral distance.

2.4 Molecular dynamics simulations

We performed molecular dynamics simulations using the LAMMPS package [7]. Our system is composed of a graphene layer folded on another graphene layer, the two of them containing 6804 atoms. The latter graphene layer was kept "frozen" during all simulations, that is, the resultant force on every atom of this layer was set to zero. Carbon atoms were modeled classically using the adaptive intermolecular reactive empirical bond order (AIREBO) potential for the C–C interaction [8]. Periodic boundary conditions were used in the y and x directions, see Figure S1, while z was finite. To model the AFM tip we use a cylinder composed of 672 Lennard-Jones particles, which were not allowed to move relative to each other. The Lennard-Jones parameters for the AFM-C interaction were ε = 1 meV and σ = 3 Å. Simulations were performed in the canonical ensemble. A Nosé–Hoover thermostat [9,10] was used in order to keep the temperature T = 300 K. The timestep used was 0.001 ps.

References

1. de Lima, A. L.; Muessnich, L. A. M.; Manhabosco, T. M.; Chacham, H.; Batista, R. J. C.; de Oliveira, A. B. *Nanotechnology* **2015**, *26* (4), 045707–045714.

2. Sader, J. E.; Chon, J. W. M.; Mulvaney, P. *Review of Scientific Instruments* **1999**, *70* (10), 3967–3969.

3. Sahin, O.; Erina, N. *Nanotechnology* **2008**, *19* (44), 445717.

4. Vasconcelos, T. L.; Archanjo, B. S.; Oliveira, B. S.; Valaski, R.; Cordeiro, R. C.; Medeiros, H. G.; Rabelo, C.; Ribeiro, A.; Ercius, P.; Achete, C. A.; Jorio, A.; Cançado, L. G. *Advanced Optical Materials* **2018**, *6* (20), 1800528.

5. Frank, O.; Tsoukleri, G.; Parthenios, J.; Papagelis, K.; Riaz, I.; Jalil, R.; Novoselov, K. S.; Galiotis, C. *ACS Nano* **2010**, *4* (6), 3131–3138.

6. Silva, D. L.; Campos, J. L. E.; Fernandes, T. F.; Rocha, J. N.; Machado, L. R.; Soares, E. M.; Miquita, D. R.; Miranda, H.; Rabelo, C.; Vilela Neto, O. P.; Jorio, A.; Cançado, L. G. *Carbon* **2020**, *161*, 181 –189.

7. Plimpton, S. *Journal of Computational Physics* **1995**, *117* (1), 1 –19.

8. Stuart, S. J.; Tutein, A. B.; Harrison, J. A. *The Journal of Chemical Physics* **2000**, *112* (14), 6472–6486.

9. Nosé, S. *The Journal of Chemical Physics* **1984**, *81* (1), 511–519.

10. Hoover, W. G. *Phys. Rev. A* **1985**, *31*, 1695–1697.
